# In Situ-Prepared Nanocomposite for Water Management in High-Temperature Reservoirs

**DOI:** 10.3390/gels11060405

**Published:** 2025-05-29

**Authors:** Hui Yang, Jian Zhang, Zhiwei Wang, Shichao Li, Qiang Wei, Yunteng He, Luyao Li, Jiachang Zhao, Caihong Xu, Zongbo Zhang

**Affiliations:** 1 Key Laboratory of Science and Technology on High-Tech Polymer Materials, Institute of Chemistry, Chinese Academy of Sciences, Beijing 100190, China; 18840163221@163.com (L.L.); 18454320579@163.com (J.Z.); caihong@iccas.ac.cn (C.X.); 2 State Key Laboratory of Offshore Oil and Gas Exploitation, Beijing 102209, China; zhangjian@cnooc.com.cn (J.Z.); wangzhw28@cnooc.com.cn (Z.W.); lishch23@cnooc.com.cn (S.L.); 3 CNOOC Research Institute Co., Ltd., Beijing 100028, China; 4 Institute of Geographic Sciences and Natural Resources Research, Chinese Academy of Sciences, Beijing 100101, China; weiq@igsnrr.ac.cn; 5 Institute of Mechanics, Chinese Academy of Sciences, Beijing 100190, China; hyt@imech.ac.cn

**Keywords:** nanocomposite, high-temperature resistance, water management

## Abstract

In the field of enhanced oil recovery (EOR), particularly for water control in high-temperature reservoirs, there is a critical need for effective in-depth water shutoff and conformance control technologies. Polymer-based in situ-cross-linked gels are extensively employed for enhanced oil recovery (EOR), yet their short gelation time under high-temperature reservoir conditions (e.g., >120 °C) limits effective in-depth water shutoff and conformance control. To address this, we developed a hydrogel system via the in situ cross-linking of polyacrylamide (PAM) with phenolic resin (PR), reinforced by silica sol (SS) nanoparticles. We employed a variety of research methods, including bottle tests, viscosity and rheology measurements, scanning electron microscopy (SEM) scanning, density functional theory (DFT) calculations, differential scanning calorimetry (DSC) measurements, quartz crystal microbalance with dissipation (QCM-D) measurement, contact angle (CA) measurement, injectivity and temporary plugging performance evaluations, etc. The composite gel exhibits an exceptional gelation period of 72 h at 130 °C, surpassing conventional systems by more than 4.5 times in terms of duration. The gelation rate remains almost unchanged with the introduction of SS, due to the highly pre-dispersed silica nanoparticles that provide exceptional colloidal stability and the system’s pH changing slightly throughout the gelation process. DFT and SEM results reveal that synergistic interactions between organic (PAM-PR networks) and inorganic (SS) components create a stacked hybrid network, enhancing both mechanical strength and thermal stability. A core flooding experiment demonstrates that the gel system achieves 92.4% plugging efficiency. The tailored nanocomposite allows for the precise management of gelation kinetics and microstructure formation, effectively addressing water control and enhancing the plugging effect in high-temperature reservoirs. These findings advance the mechanistic understanding of organic–inorganic hybrid gel systems and provide a framework for developing next-generation EOR technologies under extreme reservoir conditions.

## 1. Introduction

Waterflooding, a widely adopted technique for enhanced oil recovery (EOR), has been extensively applied for decades [1]. However, many mature oilfields now face critical challenges, including reservoir conformance issues and premature water breakthrough [2]. These problems lead to diminished sweep efficiency, elevated water cut, and increased residual oil saturation [3]. Consequently, they contribute to suboptimal economic returns from waterflooding [4], costly wastewater treatment [5], and significant environmental risks [6]. To address these inefficiencies, conformance control—a process utilizing chemical or mechanical interventions to mitigate excessive water production [7]—has emerged as a pivotal strategy for optimizing EOR performance in aging reservoirs worldwide [8].

Cross-linked polymer gels remain the most widely utilized technology for improving sweep efficiency, enhancing oil production, and controlling excessive water output in reservoir operations. Since the development of diverse cross-linking agents, the operational temperature limit of these gels has been extended to 121 °C for water shutoff treatments [9]. Additionally, hybrid gels incorporating solid particles—such as silica nanoparticles—have gained prominence due to their superior mechanical strength [10], enhanced thermal stability [11], and robust plugging performance compared to unmodified gel systems [12]. However, critical limitations persist: a short gelation time (GT) and field application constraints. For example, GT reaches 9–48 h at a temperature of 80 °C [13], decreases from 54.0 to 15.5 h with increasing temperature from 100 to 130 °C [14], or decreases from ~16 to ~13, ~11, and ~9 h when silica nanoparticles are added at concentrations of 0.10, 0.20, and 0.30 wt%, respectively [6]. Current GT profiles fail to meet the requirements of large-spacing well configurations, where extended gelation periods are essential for uniform propagation across heterogeneous reservoirs. The adjustability of composite gelation time at high temperatures is limited, primarily because the interactions between organic and inorganic components remain not fully revealed.

**Figure 1:**
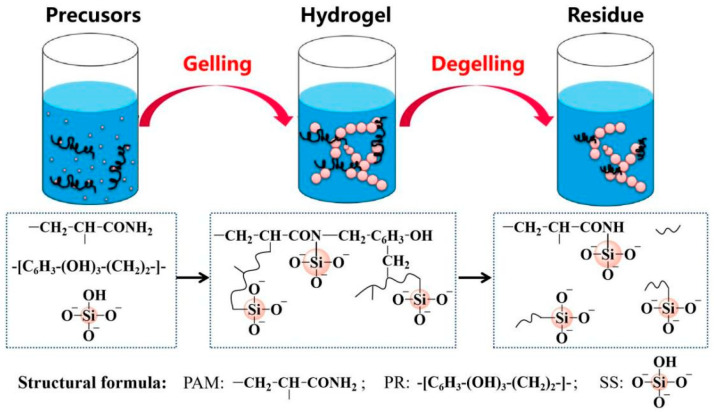
Diagrammatic representation of the gel formation and dissolution processes.

Considering the benefits of composite gels, we developed a silica-sol-reinforced PAM/phenolic aldehyde cross-linked polymer gel system (Figure 1), taking advantage of our silica sol’s pH stability in a broad weakly alkaline range [15]. As the mixed system’s pH remains mildly alkaline and changes little before and after the reaction, the inorganic component’s addition is not expected to shorten the gelation time. This hybrid formulation leverages the synergistic effects of organic–inorganic components to address field application challenges. Its key performance advantages lie in the following: (1) Extended gelation time and thermal stability—the system exhibits prolonged gelation kinetics (adaptable to large well spacing) and maintains structural integrity at elevated temperatures. (2) High operational efficiency—liquid-phase delivery (polymer solution + nanoparticle suspension) simplifies field deployment compared to solid filler-based systems, enhancing safety and scalability [16]. (3) Gel degradability—the designed complex hydrogel, which can undergo reverse chemical reactions under specific conditions to contract or break down and restore fluidity, effectively mitigates risks in the plugging process.

The drive behind this work emanates from the existing knowledge gap in silica sol nanoparticles’ role in enhancing polymer-based in situ-cross-linked gel functionality, especially in high-temperature settings. Presently, while silica sol has been sporadically utilized in gel systems, there is a paucity of in-depth studies comprehensively exploring its influence on gelation dynamics and internal water interactions, which are pivotal for high-temperature EOR applications. To address these needs, our study conducted a systematic investigation into how silica sol affects gelation time and strength. We explored how varying silica sol contents impact the gel’s thermal stability, meticulously measured the fractions of free and bound water within the gels to understand water behavior within the matrix, and employed advanced techniques to observe and analyze the gel microstructures. Based on these findings, mechanisms for silica sol’s enhancement of gel strength and thermal stability were proposed. Finally, as a typical application, the temporary plugging performance and gel-breaking response were assessed. This work establishes a tunable gel system with dual-phase reinforcement mechanisms, offering a practical solution for high-temperature reservoirs requiring delayed gelation and sustained mechanical performance.

## 2. Results and Discussion

### 2.1. Gelation Performance

**Figure 2:**
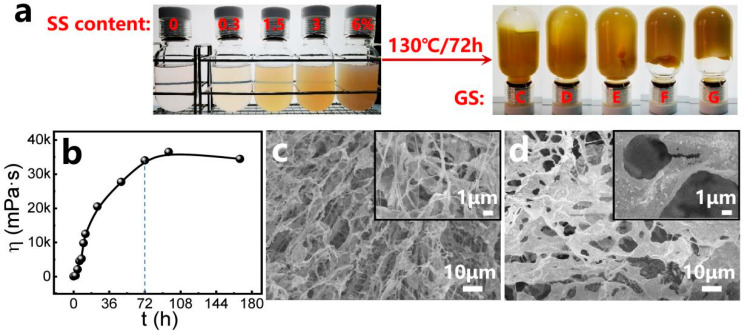
(**a**) Typical examples of composite gels, before and after gelation, at different SS contents of 0–6% and at 130 °C; (**b**) viscosity profile during different periods, at an SS content of 0.3% and at a reaction temperature of 130 °C. Micrographs of prepared gel samples: (**c**) without SS; (**d**) with 0.3% SS.

The precursor solutions containing varying concentrations of SS exhibited progressively darker coloration and were thermally cured at 130 °C. Mature composite gels formed after several days (Figure 2a), demonstrating a gradual increase in gel strength (GS) from Code C (0% SS) to Code G (6% SS). The gelation time (GT) was monitored via optical imaging (Table S1), revealing a minimum threshold exceeding 48 h.

To precisely determine GT, twelve replicates at fixed SS concentrations were prepared and analyzed under controlled thermal conditions (130 °C). Time-lapsed images (45°-angled and inverted views, Table S2) showed reduced transparency and intensified coloration starting at 2 h, with negligible morphological changes beyond 72 h despite continued color darkening. Furthermore, the viscosity profiles of gel samples prepared at varying reaction times were measured as illustrated in Figure 2b. The viscosity values exhibit a linear increase with prolonged reaction time. Near 72 h, the viscosity growth rate plateaued, confirming a gelation time of approximately 72 h. To the best of our knowledge, the in situ cross-linking composite gel system presented here exhibits the longest GT at high temperature.

SEM analysis reveals critical insights into the micromorphological evolution of mature gels with varying SS contents (Figure 2c,d). From the micrographs, three-dimensional network structures are observed, in which polymer chain bunches with a thickness of several micrometers surround the pores with a coverage of tens of micrometers (Figure 2c). In the gelling solutions, amide groups (−CONH_2_) from PAM cross-link with the hydroxyl groups (-CH_2_OH) from PR, contributing to the porous network structure [17]. The distributed pores are the main storage spaces of water in gels. After adding SS, it can be observed that silica nanoparticles aggregate together and form varied arrangements. The aggregations and arrangements of silica nanoparticles are attached to the polymer chain bunches and meshes of the gel structure (Figure 2d), which strengthens the network structure of the gel.

### 2.2. Surface Modification

**Figure 3:**
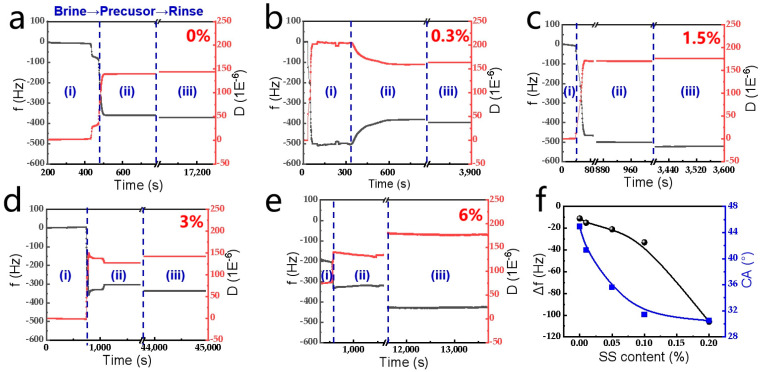
(**a**–**e**) Frequency and dissipation shift as a function of the adsorption time in the presence of different SS contents; (**f**) the wettability of the adsorbed surfaces in the presence of different SS contents.

The in situ adsorption/desorption dynamics of mixed precursor solutions containing SS on silica surfaces was analyzed via quartz crystal microbalance with dissipation monitoring (QCM-D), tracking real-time shifts in frequency (Δf) and dissipation (ΔD) (Figure 3). The experiments were performed as follows (three main stages ((i)~(iii)) can be distinguished, with Figure 3a as an example): (i) Baseline stabilization: the quartz crystal sensor was equilibrated in formation brine to establish initial Δf and ΔD values. (ii) Precursor adsorption: the introduction of SS-containing solutions triggered abrupt Δf and ΔD decreases, attributed to the rapid surface adsorption of PAM, PR molecules, and SS nanoparticles. (iii) Desorption phase: the reintroduction of brine removed loosely bound molecules once adsorption saturation (Δf or ΔD plateau) was achieved.

It can be concluded that the adsorption process of the mixed precursor systems onto the solid surfaces (Figure 3b–e) is similar to that of the organic precursor system (Figure 3a). In addition, Δf decreases with the increase in SS content, which generally suggests an increase in the adsorbed mass. This indicates that SS prefers to adsorb on SiO_2_’s surface or facilitates the adsorption of the organic precursor. CA measurements reveal improved surface hydrophilicity, decreasing from 44.9° to 30.5° with rising SS content (Figure 3f), aligning with the goal of reservoir wettability modification. This SS-driven hydrophilicity enhancement suggests potential for tailoring gel–surface interactions in reservoirs.

### 2.3. Enhancement Properties and Mechanisms

**Figure 4:**
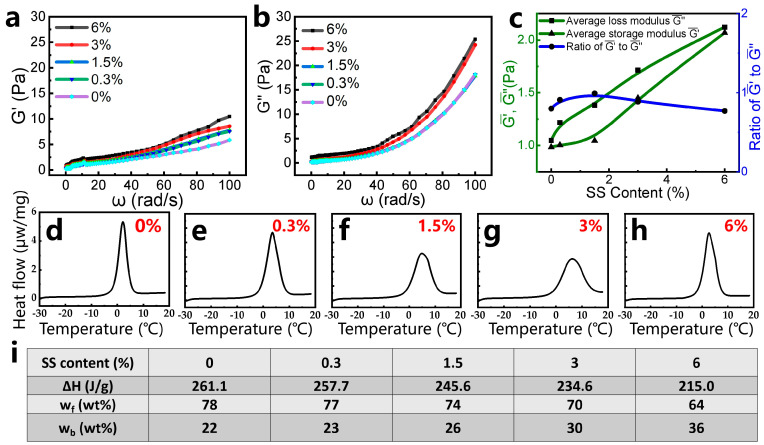
(**a**) The storage modulus (G′) and (**b**) loss modulus (G″) of gel samples prepared with different SS contents, within the frequency range from 0.1 rad/s to 100 rad/s. (**c**) Average storage modulus ($\bar{G\prime }$) and average loss modulus ($\bar{G\prime \prime }$), within the frequency range from 0.1 rad/s to 100 rad/s. (**d**–**h**) DSC curves of the gel samples prepared with different SS contents of 0, 0.3, 1.5, 3, and 6%; the scanning temperatures range from −30 to 20 °C. (**i**) Mass fractions of free water and bound water in the gel samples.

To elucidate the correlation between viscoelastic properties and SS content, frequency-dependent rheological analyses were conducted on the mature gels (Figure 4a,b). Both storage modulus (G′) and loss modulus (G″) increase monotonically across the tested frequency range (0.1–100 rad/s) as the SS content rises from 0% to 6%. The average $\bar{G\prime }$ and $\bar{G\prime \prime }$ values exhibit a linear dependence on SS loading (Figure 4c), confirming enhanced elastic and viscous contributions from SS incorporation. At equivalent shear rates, G″ consistently exceeds G′, indicating a viscosity-dominated response characteristic of weakly cross-linked or liquid-like gel systems [18]. The amplified viscoelasticity aligns with macroscopic gel strength trends (Figure 4a), where SS nanoparticles act as multifunctional cross-linkers to improve network rigidity while maintaining dynamic fluidity.

In cross-linked polymer gels, water exists in two distinct states: free water (mobile, unbound) and bound water (immobilized via polymer–water interactions) [6]. Differential scanning calorimetry (DSC) was employed to quantify these water states in gels containing varying SS contents, with the following observations. The solid–liquid phase transition of free water occurs at about 0 °C. So, when the samples are heated gradually near 0 °C, the heat absorption is caused by the melting of frozen free water. Meanwhile, the solid–liquid transition of bound water occurs at temperatures away from 0 °C [19], because of the strong interactions between bound water and polymer molecules [20]. DSC measurements were applied to investigate the content of different types of water within the gels prepared with different contents of SS. In this part, the scanning temperature ranged from −30 to 20 °C and all of the gel samples fully gelled. As shown in Figure 4d–h, single endothermic peaks appear at about 0–10 °C, attributable to the melting of frozen free water in the gel samples.

By the integration of heat flow with time, the endothermic enthalpies required to melt the free water during the DSC scanning process were calculated. On the basis of the melting enthalpy of free water, the mass fractions of free water and bound water in the gel samples can be obtained from the following formulas [6]:

w_f_ = ΔH/ΔH_0_ (1)

w_b_ = 1 − w_f_ (2)

where w_f_ and w_b_ are the mass fractions of free water and bound water, respectively (%); ΔH is the enthalpy (J/g); and ΔH_0_, with a constant value of 333.5 J/g, is the melting enthalpy of free water (J/g).

The thermal characteristics and water distribution in the gel samples are quantified in Figure 4i, revealing two key trends: (1) Water state modulation by SS content—A progressive decrease in free water’s endothermic enthalpy and a concomitant increase in the bound water mass fraction (23–36% vs. 22% in SS-free gel) are observed with elevated SS content (0.3–6%). (2) The hydrophilic mechanism of silica nanoparticles—The hydroxyl-rich surfaces of SS nanoparticles enhance water retention via hydrogen bonding, directly increasing bound-water proportions [21]. This aligns with the improved hydrophilic behavior demonstrated in Figure 3f. (3) Thermal stability implications—Elevated bound water content reduces dehydration susceptibility, suggesting enhanced thermal resilience in SS-modified gels [6]. Additionally, we conducted thermogravimetric analysis (TGA) to evaluate the thermal stability of gels with varying silica nanoparticle (SS) contents (0–6 wt%). As demonstrated in Figure S1a, the aging temperature of the pure gel (0% SS) is 120 °C, with a residual mass of 0.4% corresponding to hydrate water loss. Intriguingly, increasing the SS content to 0.3–6% elevates the aging temperature to 135–148 °C, while simultaneously reducing the residual mass to 2.4–10.9% (Figure S1b–e). Furthermore, the final mass loss at 1000 °C exhibits a clear SS-dependent trend, increasing from 0% (pure gel) to 1.2–9.0% for composite gels. These results indicate that SS incorporation enhances thermal resistance during the aging phase but accelerates decomposition at high temperatures, due to organic–inorganic interactions.

**Figure 5:**
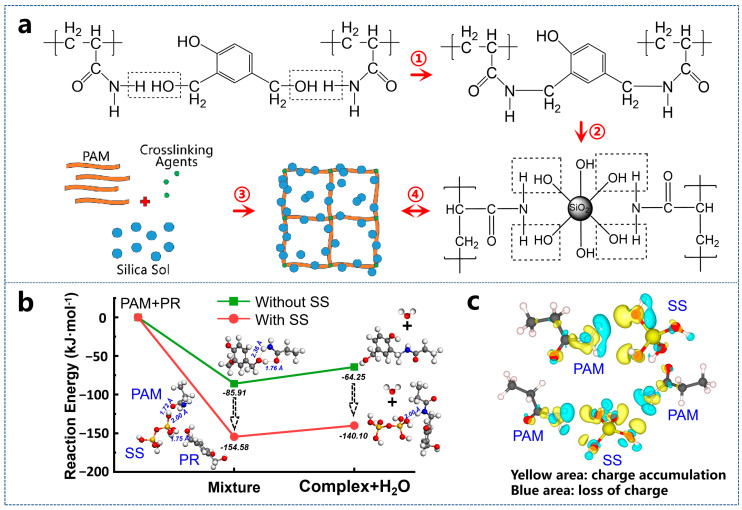
(**a**) Strengthening mechanism of silica nanoparticles in the gel network; (**b**) calculation of binding energy between PAM and SS molecules; (**c**) charge distribution analysis of the intermolecular interactions.

In the PAM/water-soluble PR system, hydroxyl groups (from PR) undergo co-condensation with amide groups (from PAM), forming C−N bonds (Figure 5a ①) that facilitate the development of a three-dimensional organic gel network [22]. The incorporation of silica nanoparticles into the gelling solution further enhances two aspects of this structural framework: gel strength and thermal stability. As illustrated in Figure 5a ②–④, silica nanoparticles aggregate into arrangements during the cross-linking process. By setting the initial energy of isolated reactants PAM and PR to zero, computational analysis reveals distinct reaction pathways with and without SS (Figure 5b). In the absence of SS, weak hydrogen bonding occurs between the –OH group of PAM and the carbonyl oxygen of PR (O–H bond length: 1.76 Å), resulting in a system energy reduction to −85.91 kJ·mol^−1^. The subsequent release of one H_2_O molecule generates a complex, accompanied by an energy increase of 21.66 kJ·mol^−1^. In contrast, the presence of SS enables simultaneous dual hydrogen bonding between the Si–OH cluster and both PAM and PR, significantly lowering the total system energy to −154.58 kJ·mol^−1^—a thermodynamically favorable pathway. The strengthened interfacial interactions between Si–OH, PAM, and PR reduce the energy barrier for complex formation, yielding a smaller energy increase of 14.48 kJ·mol^−1^. Furthermore, stabilizing interactions between the complex and the Si–OH cluster maintain the system energy at −140.10 kJ·mol^−1^, demonstrating the catalytic role of SS in facilitating the organic–inorganic reaction and promoting long-chain molecular assembly.

Additionally, the binding energy between -NH_2_ groups and the Si_2_-OH cluster is stronger than that with Si-OH (Figure S2), indicating that hydrogen bonding facilitates silica particle growth. The oxygen atom acquires a partial negative charge due to its stronger electronegativity in attracting shared electrons, while the hydrogen atom bears a partial positive charge. This charge asymmetry generates Coulomb attraction between O and H, forming a weak hydrogen bond (Figure 5c). These structured assemblies integrate into the polymer chain clusters and meshes, significantly improving the gel’s mechanical integrity. The resultant structural reinforcement leads to a marked increase in gel strength. This mechanistic interpretation is corroborated by FTIR spectroscopy (Figure S3). The broad absorption at 3300 cm^−1^ integrates N–H (PAM), O–H (PR), and Si–OH (SS) stretching vibrations, with peak broadening indicative of extensive hydrogen bonding networks [23]. The amide I band at 1637 cm^−1^ (C=O stretching) and aromatic C=C vibrations (PR) confirm organic phase integrity [24], while the blue-shifted Si–O stretching peak at 1128 cm^−1^ (Δ*ν* = +28 cm^−1^ from 1100 cm^−1^) directly evidences interfacial hydrogen bonding between organic components and the silica network [25]. Critically, the progressive intensity enhancement of the 1128 cm^−1^ peak with increasing SS content quantifies the concentration-dependent strengthening of organic–inorganic interactions, aligning with computational predictions. Collectively, these results establish hydrogen bonding as the dominant interfacial linkage mechanism, synergistically reinforced by SS to optimize composite gel performance.

The thermal stability enhancement is driven by two synergistic interactions: hydrogen bonding and electrostatic interactions [26]. Surface hydroxyl (-OH) groups on silica nanoparticles form hydrogen bonds with water molecules, converting free water into bound water [27]. Negatively charged silica nanoparticles attract hydronium ions (H_3_O^+^) through electrostatic forces, further stabilizing the bound water phase [28]. The combined effects yield a higher bound water ratio compared to silica-free gels, correlating with stronger hydrophilicity, superior water retention, and enhanced thermal resistance [29].

In the study of gelation kinetics for organic–inorganic hybrid hydrogels, silica sol systems have a longer gelation time than those with directly added silica nanoparticles. This is due to the following reasons: the nanoparticles in silica sols are highly pre-dispersed, giving the system excellent colloidal stability, and the system’s pH stays stable at 8–9 throughout the gelation process. This allows it to stay in a metastable, non-equilibrium liquid-phase state for a long time. The homogeneous dispersion of nanoparticles affects reaction kinetics in two main ways: (1) The suppression of local concentration gradients: Pre-dispersed particles eliminate microscopic concentration fluctuations caused by agglomeration, reducing the driving force for heterogeneous nucleation [30]. (2) Dynamic interfacial equilibrium: Uniformly exposed hydroxyl groups establish adsorption–desorption equilibrium at reaction interfaces, slowing down condensation reactions [31].

In contrast, exogenous silica nanoparticles, limited by intrinsic factors such as surface hydroxyl passivation and insufficient dispersion, are prone to forming discrete “reactive hotspots” within organic matrices. These heterogeneous microdomains facilitate localized condensation via autocatalytic mechanisms, resulting in premature gel network locking and macroscopic phase separation.

The delayed gelation behavior of silica sol systems fundamentally originates from their distinctive multi-scale synergistic effects: Colloidal stability ensures sustained particle dispersion through electrostatic repulsion and steric hindrance, while controlled condensation kinetics enables self-regulated reaction rates via the gradual consumption of surface hydroxyl groups. This spatiotemporally ordered cross-linking process establishes an optimal thermodynamic window for the hierarchical assembly of three-dimensional networks, and it is thereby expected to achieve the synergistic optimization of structural homogeneity, mechanical integrity, and functional tunability.

### 2.4. Application Performance

#### 2.4.1. Aging Resistance

**Figure 6:**
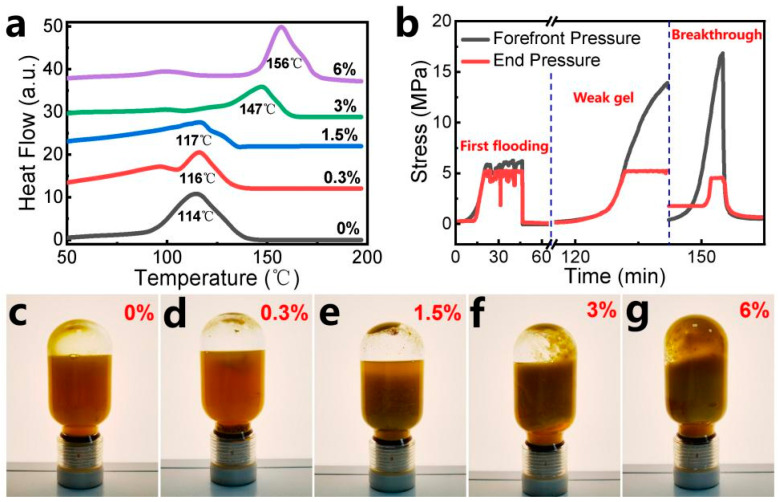
(**a**) DSC curves of hydrogels with different SS contents of 0~6%; (**b**) conductivity of PAM the hydrogel system containing 1% silica sol; (**c**–**g**) mature gels with different SS contents of 0, 0.3, 1.5, 3, and 6% aging for 60 days at 130 °C.

The thermal resistance of hydrogels with varying silica sol contents (0% to 6%) was systematically evaluated using DSC measurements. During testing, the temperature was ramped from 50 °C to 200 °C, with the peak endothermic rate (indicating the maximum water loss rate) identified as the sharpest endothermic peak in the DSC thermograms (Figure 6a). Below the temperature inflection point, heat absorption increases steadily with rising temperature due to the gradual evaporation of free and bound water within the hydrogel matrix. Above the inflection temperature, the heat flow curve declines sharply, signifying near-complete dehydration of the hydrogel. This structural dehydration correlates with irreversible damage to the hydrogel network. The incorporation of nanosilica sol significantly enhances the thermal resistance. The maximum tolerable temperature rises from 114 °C (0% SS) to 156 °C (6% SS). 

Further evaluation demonstrated the robust stability of the hydrogel under prolonged thermal stress, which was performed as shown in Figure 6c–g. After 60 days of high-temperature exposure, the hydrogels exhibited negligible alterations in macroscopic appearance, indicating structural integrity. Less significant syneresis (water expulsion) was observed, confirming effective water retention within the gel matrix. The viscosity retention rate of the hydrogel systems exceeded 90%, validating the material’s resistance to thermal degradation and sustained rheological performance. As the SS content increased, the hydrogels exhibited a more cohesive texture and a higher viscosity retention rate. The improvement stems from the reinforcement effect of SiO_2_ nanoparticles: mechanical strengthening and water retention, i.e., silica particles fortify the hydrogel skeleton—reducing thermal degradation—and nanoparticles act as anchoring sites for water molecules, improving hydration stability.

#### 2.4.2. Practical Application

The simulated core conductivity experiments provide dual insights: (1) Injection feasibility—assessing the injectability of hydrogel systems in real reservoir formations. (2) Plugging efficiency—evaluating the hydrogel’s capacity to block fluid pathways within the core. The hydrogel system containing 0.3% SS demonstrates effective advancement within the core. Due to the weak gelation capacity of the low-strength weak gel system, it exhibits favorable deep migration and transport capabilities under the subsequent flushing force of water flooding. During the initial water flooding stage, the pressure difference between the front and tail ends of the system measured 0.6 MPa. After 5 days of curing, this pressure difference significantly increased to 12.31 MPa (Figure 6b). As quantified by Equation (4) (Section 4.3.9), the core permeability exhibited a reduction of approximately 92.4% between pre- and post-plugging conditions. The system achieves superior sealing performance compared with in situ-cross-linked polymer gels [32].

#### 2.4.3. Gel Degradation

**Figure 7:**
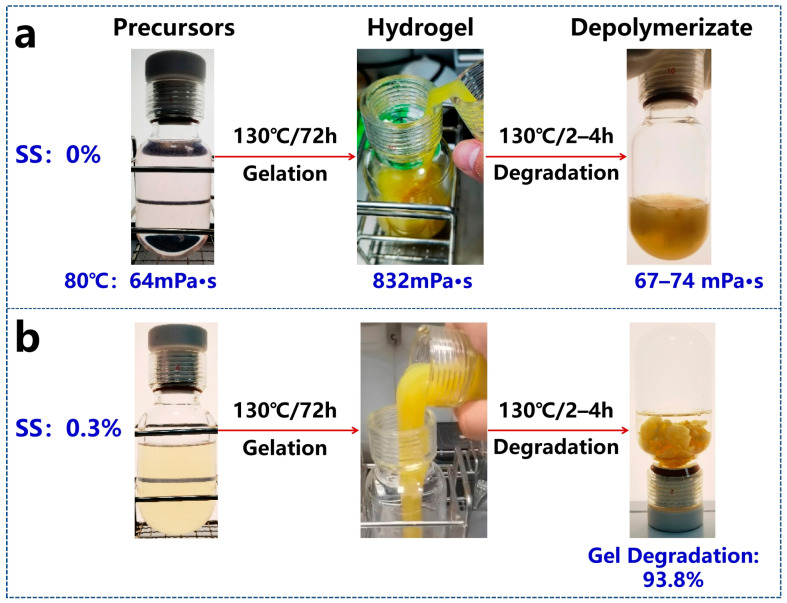
Gel breaking process of the composite gel with (**a**) 0% SS and (**b**) 0.3% SS.

The 10% GB solution was introduced into the gelled composite system maintained at 130 °C. Within 30 min of thermal treatment, the gel system exhibited initial degradation with visible structural heterogeneity (Figure 7). Complete degradation was achieved after 150 min, demonstrating a weight loss ratio of 93.8% (calculated as follows: [Initial weight − Residual weight]/Initial weight [33]). This degradation behavior confirms that the gelled composite system retains the organic component’s degradability, enabling conventional methods to effectively mitigate plugging risks while maintaining temperature-responsive functionality [34]. Notably, the synergistic organic–inorganic network structure ensures controlled decomposition without blockage risk.

## 3. Conclusions

This study systematically investigates a silica-reinforced cross-linked polymer gel, focusing on its gelation behavior, thermal resilience, structural reinforcement mechanism, and related applications. The key findings are as follows:

(1) Gelation Performance Enhancement: Silica sol extended the gelation time to 72 h while significantly improving gel strength, attributed to the structured assemblies integrate into the polymer chain clusters and meshes. Both storage and loss modulus increased compared to silica-free gels, confirming mechanical reinforcement via hybrid organic–inorganic networks.
(2) Thermal Stability and Water Retention: Bound water content rose from 22% (SS-free) to 36% in SS-modified gels, correlating with hydroxyl-mediated hydration layers on nanoparticle surfaces. The maximum tolerable temperature rose from 114 °C (0% SS) to 156 °C (6% SS), owing to stabilized water–polymer interactions and restricted chain mobility.
(3) Strengthening Mechanism: A three-dimensional network architecture was observed, where silica aggregates form an interfacial interaction with polymer bundles, mechanically reinforcing the gel matrix. The synergistic interplay of hydrogen-bonded hydration layers and electrostatic interactions elevates the bound-water ratio, correlating with stronger hydrophilicity, superior water retention, and enhanced thermal resistance.
(4) Field-Relevant Performance: The core permeability reduction of 92.4% post-gelation demonstrates effective pore throat sealing, critical for water management in fractured reservoirs. The synergistic network design enables controlled decomposition without secondary contamination, addressing environmental concerns in oilfield applications.

This work establishes a framework for designing nanoparticle-enhanced gels with tunable rheological and thermal properties for harsh reservoir conditions.

In terms of future research directions, the following proposals can be made:

(1) Further exploration of the hydrogel’s properties under different dehydration conditions to better understand the correlation between structural dehydration and network damage.
(2) Investigation of ways to enhance the hydrogel’s stability and reversibility under varying environmental conditions.
(3) Exploration of hydrogels’ potential applications in specific fields to validate their practical utility.

## 4. Materials and Methods

### 4.1. Materials

The polyacrylamide (PAM) with an average molecular weight of 12,000,000 g/mol, phenolic resin (PR) with a weight average molecular weight of 11,411 g/mol, and the gel breaker (GB) were provided from the laboratory of CNOOC. The inorganic precursor of silica sol (SS, 30%), with a narrow particle size distribution of about 50.0 nm, was prepared in our laboratory [15]. Sodium chloride, potassium chloride, calcium chloride, magnesium chloride hexahydrate, sodium sulfate, and sodium bicarbonate were AR-grade materials. The synthetic formation brine used contained 2323.52 mg/L Na^+^, 26.57 mg/L K^+^, 115.53 mg/L Ca^2+^, 52.29 mg/L Mg^2+^, 3689.88 mg/L Cl^−^, 11.16 mg/L SO_4_^2−^, and 619.22 mg/L HCO_3_^−^.

### 4.2. Method for Gel Preparation and Gel Degradation

#### 4.2.1. Gel Preparation

Using the synthetic formation brine, the silica sol was diluted to the concentration of 0%, 0.3%, 1.5%, 3%, and 6%, respectively. We set the stirring speed to 200RPM in the diluted silica sol, and kept the temperature at 45 °C. The high-purity nitrogen was aerated to the system at a flow rate of 10mL/min. The PAM powder was added into the system little by little. Two hours later, the mixed system was cooled down to room temperature. A certain amount of PR was added into the mixed system at a stirring speed of 200 RPM for 15min. The concentrations of PAM and PR were both set to 2500 mg/L. The precursor solution was degassed by purging with high-purity nitrogen (99.999%) at 1 L/min for 30 s, followed by static settlement in a sealed flask for 12 h to allow bubble removal. Prior to sealing, a final nitrogen purge was applied to establish an oxygen-free atmosphere, ensuring stability for subsequent reactions. The sealing precursor solution was placed in an oven at a temperature of 130 °C as an example, for a period of time until a mature gel was formed.

#### 4.2.2. Gel Degradation

After the formation of a mature gel, GB at a concentration of 10% was added into the gel system at 130 °C, for a period of time until the gel was broken.

### 4.3. Characterization

The experimental workflow is depicted in Figure S4.

#### 4.3.1. Measurement of the Gelation Time (GT) and Strength (GS)

The gelation time and strength of the gel were measured through bottle tests and evaluated by the gel strength code. As introduced in a previous report [35], Codes A through I are described as follows: Code A—no detectable continuous gel formed; Code B—highly flowing gel; Code C—flowing gel; Code D—moderately flowing gel; Code E—barely flowing gel; Code F—highly deformable non-flowing gel; Code G—moderately deformable non-flowing gel; Code H—slightly deformable non-flowing gel; and Code I—rigid gel. For water shutoff gel, the initial gelation time is usually considered the period of time when the gelation solution in Code A turns to flowing gel in Code C. The frequency of gel code determination is every 2–24 h.

#### 4.3.2. Viscosity and Rheology Measurements

Viscosity measurements of the nanocomposite during the growth process were performed using a modular compact rheometer (MCR-302, Anton Paar, Physica, Graz, Austria). The test temperature was 80 °C and shear rates ranged from 0.1 to 1000 s^−1^. The rheological properties of the gelling solution and mature gel were measured at a shear rate of 200–0.1 s^−1^.

#### 4.3.3. Scanning Electron Microscopy (SEM) Scanning

The morphology features of different samples were analyzed by SEM (S-4800, HITACHI, Tokyo, Japan), operating at an accelerating voltage of 10 kV and at an electric current of 10 μA. The prepared gels were frozen to −110 °C using liquid nitrogen and metal coated for the later observation.

#### 4.3.4. Calculation Methods

To investigate the interaction between SiO_2_ nanoparticles and -NH_2_ units of PAM, we conducted density functional theoretical (DFT) calculations using VASP (Vienna Ab initio Simulation Package) [36]. The projector-augmented wave pseudopotential (PAW) method was employed to describe the interaction between ionic cores and valences [37]. The exchange–correlation effects were calculated within the generalized gradient approximation (GGA) using the Perdew−Burke−Ernzerhof (PBE) functionals, with a cutoff energy of 400 eV for the plane-wave basis set [38]. The convergence criteria for ionic and electronic calculations were set to 0.02 eV Å^−1^ and 10^−5^ eV, respectively [39]. The optimization of Si_n_-OH clusters and -NH_2_ units was performed in a cubic box of 30 Å in each direction. The Brillouin zone integration was carried out using a single k-point at the Γ point.

The binding energy (∆*E*_b_) was calculated by the following equation:

∆*E*_b_ = ∆*E* − ∆*E*_Si-OH_ − ∆*E*_-NH2_ (3)

where ∆*E* is the total electronic energy of the complex. ∆*E*_Si-OH_ and ∆*E*_-NH2_ refer to the energies of Si_n_-OH cluster and -NH_2_ unit, respectively. Bader charge analysis was conducted to investigate the charge transfer between these two species [40].

#### 4.3.5. Differential Scanning Calorimetry (DSC) Measurement

DSC measurements of the gel samples were conducted by a differential scanning calorimeter (STA 449 F3, Netzsch, Selb, Germany). In each measurement process, the gel sample, measuring about 30~45 mg, was placed in a crucible with a nitrogen purge rate of 50 cm^3^/min. The temperature change rates were 5 °C/min for all measurements. In the high-temperature test, the scanning temperature range was 50–200 °C, and the scanning temperature range was −30–20 °C when the proportion of bound water was measured.

#### 4.3.6. Quartz Crystal Microbalance with Dissipation (QCM-D) Measurement

QCM-D measurements were conducted to measure the interaction between precursors and rock surfaces. The resulting frequency difference Δ*f* is inversely proportional to the adsorbed mass [41]. The injection sequence of each sample was as follows: formation water ⇒ precursor solution ⇒ formation water ⇒ distilled water rinsing, in which the SS contents were 0%, 0.3%, 1.5%, 3%, and 6%. Commercial SiO_2_ sensors (QSX 303, Biolin Scientific, Solna, Sweden) were selected to simulate reservoir rock surfaces for the QCM-D experiments.

#### 4.3.7. Contact Angle (CA) Measurement

The water contact angles of the adsorbed surfaces were obtained using a DSA 100 (Kruss DSA CA goniometer, Hamburg, Germany) drop shape analysis system, and 3 mL of distilled water was used.

#### 4.3.8. Injectivity and Temporary Plugging Performance Evaluation

The injectivity of the gel precursor was evaluated using the experimental setup shown in Figure S5. An artificial core with a permeability of ~100 mD was saturated with formation brine and connected to the apparatus. The injection process involved three sequential phases:

(1) Initial water flooding: Brine solution was injected at 0.5 mL/min and 90 °C, with intermediate container 3 open and container 4 closed. Injection rates and nodal pressures (front/middle sections) were recorded until pressure stabilization.
(2) Gel precursor injection: Container 3 was closed, and container 4 was opened to inject the gel precursor solution. Nodal pressures were monitored to assess flow resistance during precursor delivery.
(3) Post-flooding evaluation: Brine solution was reinjected to measure residual injectivity, with pressure data collected until equilibrium.

#### 4.3.9. Plugging Performance Evaluation

Plugging efficiency was quantified through a three-stage protocol:

(1) Baseline brine flooding: The brine-saturated core underwent brine injection (container 3 open, container 4 closed) until stable nodal pressures were achieved.
(2) Gel formation: The gel precursor was injected, followed by a 3-day aging period to enable in situ cross-linking.
(3) Post-treatment evaluation: Water flooding resumed until pressure stabilization, and the plugging rate (%) was calculated using the following equation [42]:

(4) $$K=\frac{QL\mu }{A\Delta P}$$

*Q* is the medium flow rate (cm^3^/s) passing through the core under pressure difference Δ*P*; *A* is the cross-sectional area of the core (cm^2^); *L* is the length of the core (cm); *μ* is the viscosity of the injected medium fluid (mPa·s); Δ*P* is the pressure difference between the front and back in the direction of core length (10^−4^ MPa); and *K* is the proportional coefficient, i.e., the absolute permeability (*D*) of the porous medium.

## Data Availability

The data presented in this study are available on request from the corresponding authors.

## Supplementary Materials

The following supporting information can be downloaded at: https://www.mdpi.com/article/10.3390/gels11060405/s1, Figure S1: TGA and DTG curves of mature gel with different SS content of (a) 0%, (b) 0.3%, (c) 1.5%, (d) 3%, (e) 6%; Figure S2: Calculated binding energies between -NH_2_ with Si-OH (with one Si species) and Si_2_-OH (with two Si species) clusters; Figure S3: (a) FTIR spectra and (b) magnified spectral region (500–1500 cm^−1^) of composite gels with varying SS contents (0–6%); Figure S4: The flow chart of the experiment; Figure S5: Setup diagram for plugging evaluation experiment; Table S1: Optical images (placing upright, at a fixed angle of 45°, and upside down) during gelation process at different SS content and 130 °C; Table S2: Optical images (at a fixed angle of 45° and upside down) during gelation process, at the SS content of 0.3% and at 130 °C.
