# Supplementary material for: In Situ-Prepared Nanocomposite for Water Management in High-Temperature Reservoirs
Source: Gels. 2025 May 29;11(6):405. doi: 10.3390/gels11060405 (PMC12192235; doi:10.3390/gels11060405)
Supplement: Supplementary file 1 [file gels-11-00405-s001.zip › gels-3611867-supplementary.pdf]

In-situ Prepared Nanocomposite for Water Management in High-Temperature Reservoirs

Hui Yang[1,*], Jian Zhang[2,3], Zhiwei Wang[2,3], Shichao Li[2,3], Qiang Wei[4], Yunteng He[5], Luyao Li[1], Jiachang Zhao[1], Caihong Xu[1], Zongbo Zhang[1,*]

[1]Key Laboratory of Science and Technology on High-Tech Polymer Materials, Institute of Chemistry, Chinese Academy of Sciences, Beijing 100190, China

[2]State Key Laboratory of Offshore Oil and Gas Exploitation, Beijing 102209, China

[3]CNOOC Research Institute Co., Ltd., Beijing 100028, China

[4]Institute of Geographic Sciences and Natural Resources Research, Chinese Academy of Sciences, Beijing 100101, China

[5]Institute of Mechanics, Chinese Academy of Sciences, Beijing 100190, China

Characterization. TGA. Thermal gravimetric analysis (TGA) measurements were carried out to investigate the thermal decomposition process of the gel by a TA Instrument (STA449F3, NETZSCH, Germany). The temperature was scanned from 30 to 1000 °C at 10 °C/min. The test was performed in a nitrogen atmosphere with a gas flow rate of 100 mL/min.

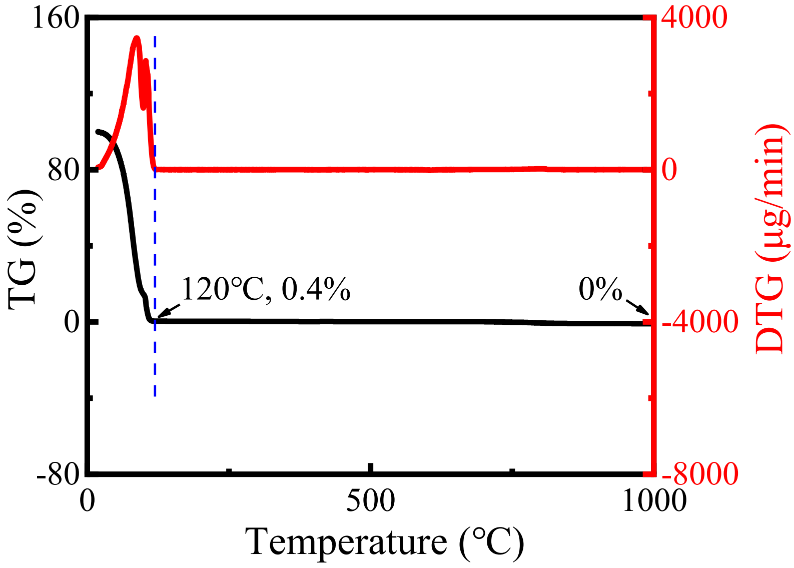

(a) pure gel with SS content of 0%

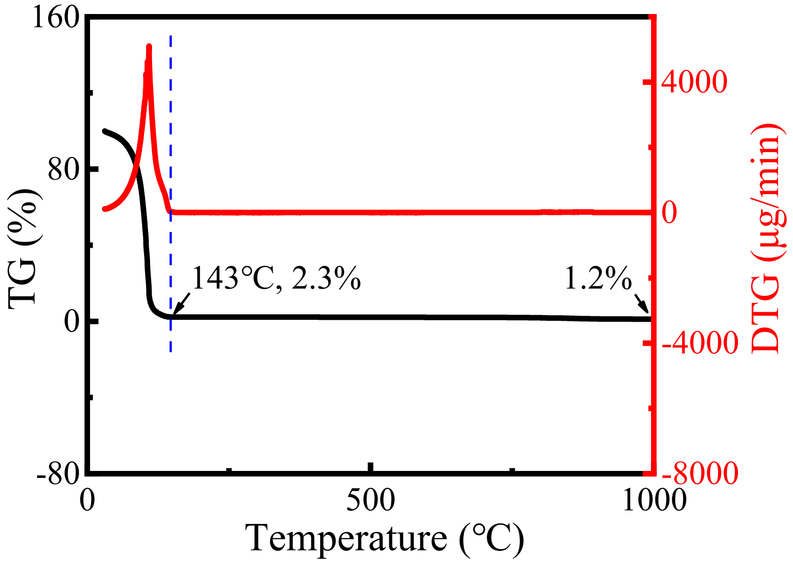

(b) composite gel with SS content of 0.3%

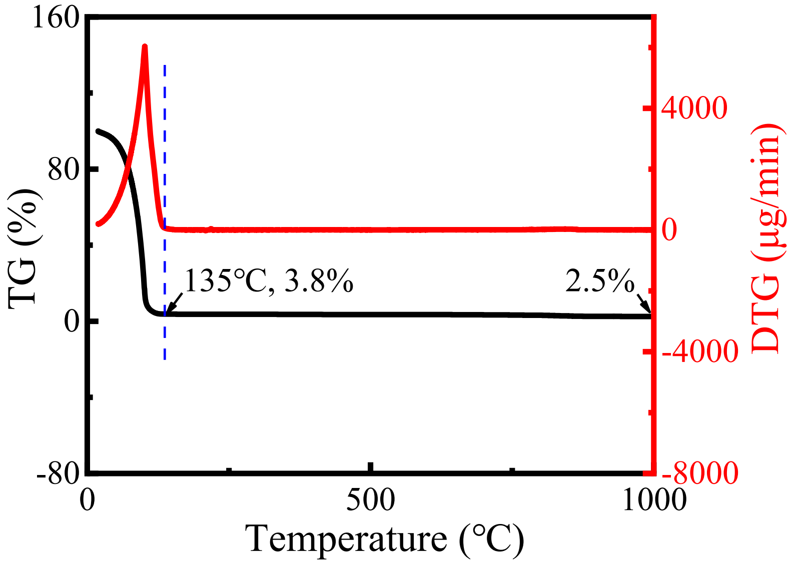

(c) composite gel with SS content of 1.5%

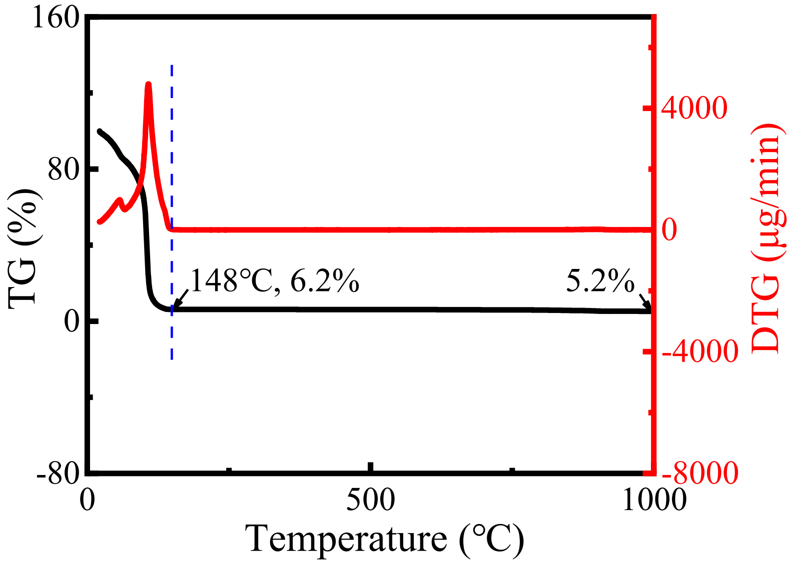

(d) composite gel with SS content of 3%

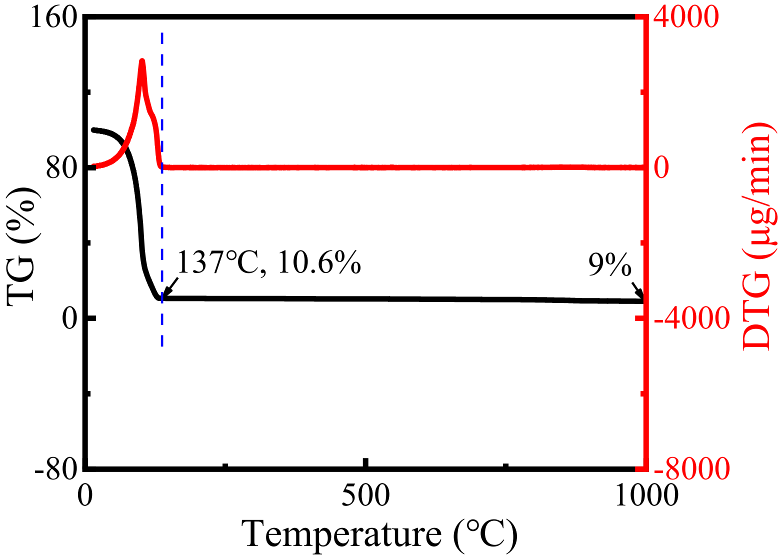

(e) composite gel with SS content of 6%

Figure S1. TGA and DTG curves of mature gel with different SS content of (a) 0%, (b) 0.3%, (c) 1.5%, (d) 3%, (e) 6%.

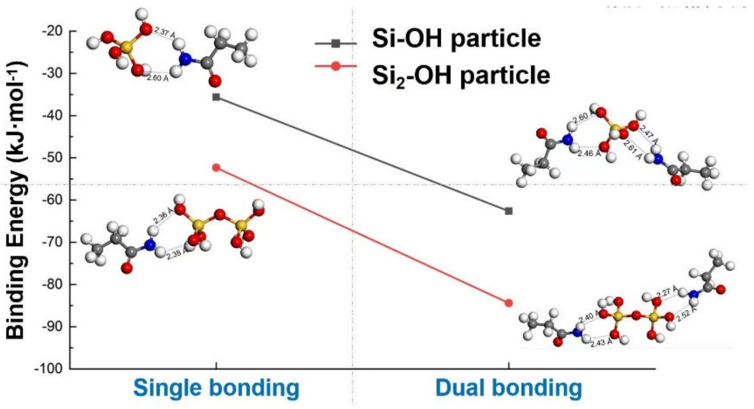

Figure S2. Calculated binding energies between -NH_2 with Si-OH (with one Si species) and Si_2-OH (with two Si species) clusters.

Characterization. FTIR. The chemical structure of the mature gels was characterized by Fourier transform infrared spectrometry (FTIR, Bruker ALPHA II, Germany). The liquid samples to be measured were placed into the ATR sample cell. The blank sample cell was used as the reference for the spectra, and the test range was 4000~500 cm^{-1} with 32 scans.

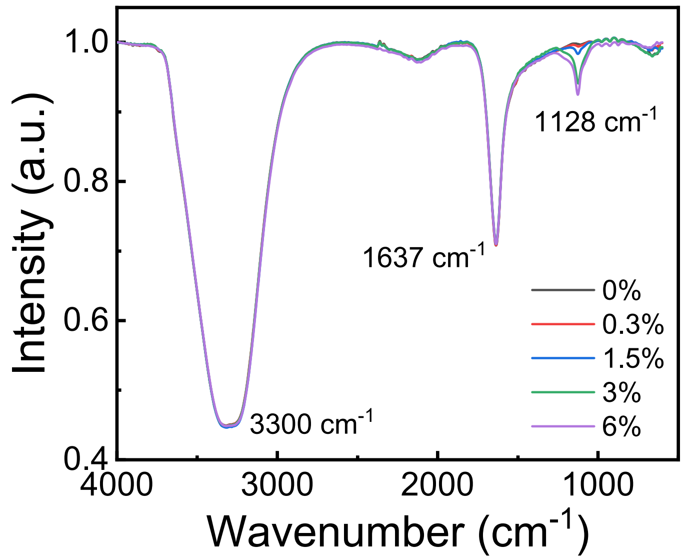

(a)

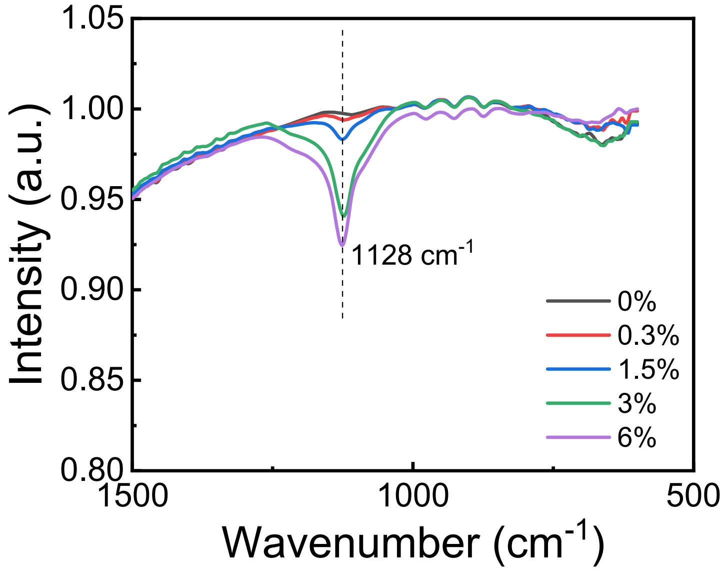

(b)

Figure S3. (a) FTIR spectra and (b) magnified spectral region (500-1500 cm^{-1}) of composite gels with varying SS contents (0-6 %).

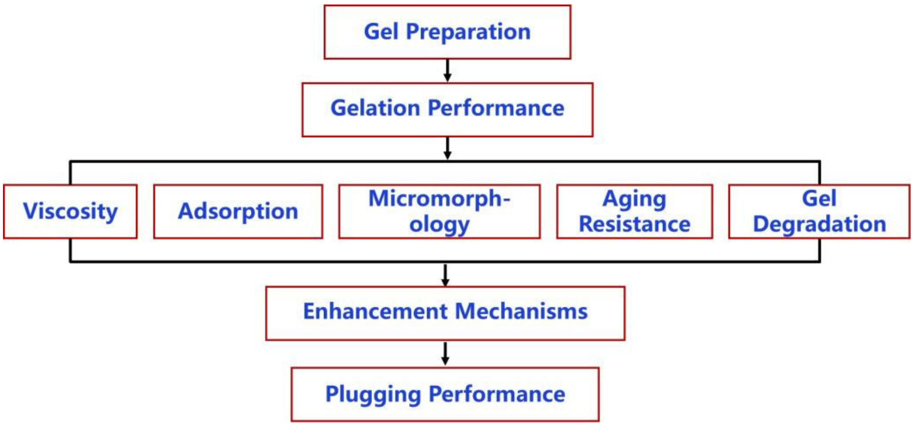

Figure S4. The flow chart of the experiment.

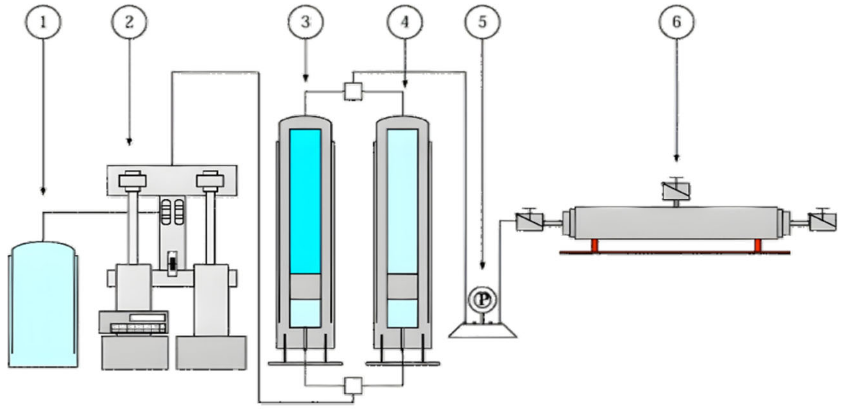

① Intermediate container - formation brine; ② High pressure pump with constant speed and constant pressure; ③ Intermediate container - formation brine; ④ Intermediate container - precursor solution; ⑤ Pressure gauge with high precision; ⑥ Artificial homogeneous core.

Figure S5. Setup diagram for plugging evaluation experiment.

Table S1. Optical images (placing upright, at a fixed angle of 45°, and upside down) during gelation process at different SS content and 130℃.

SS content / Time	0%	1%	5%	10%	20%
0h	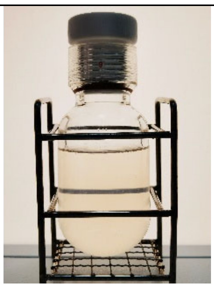	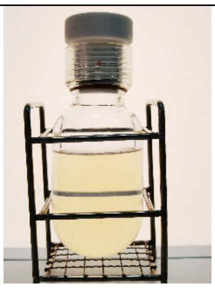	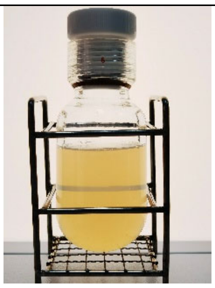	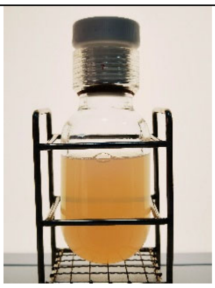	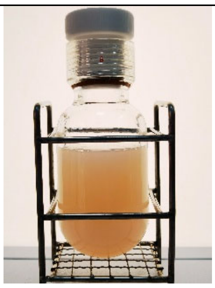
2h	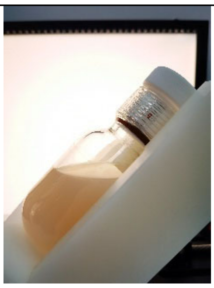	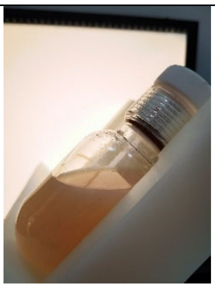	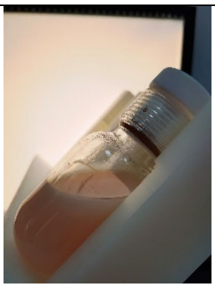	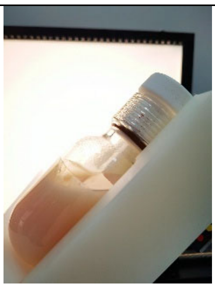	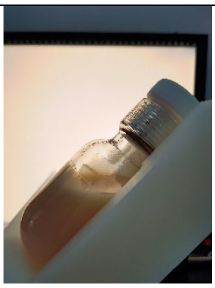
4h	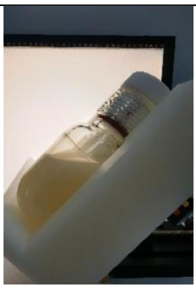	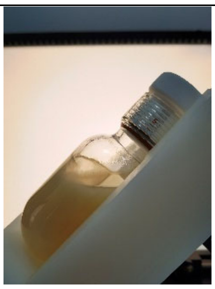	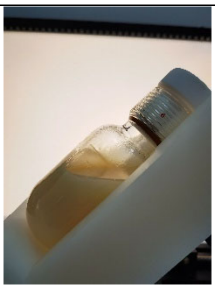	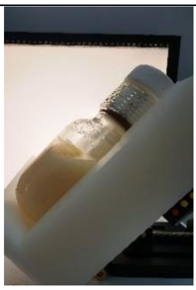	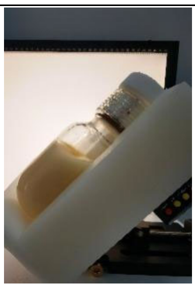
6h	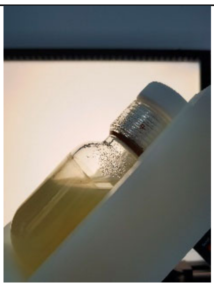	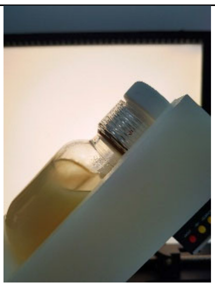	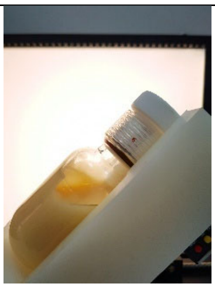	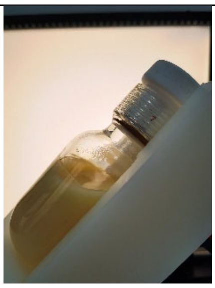	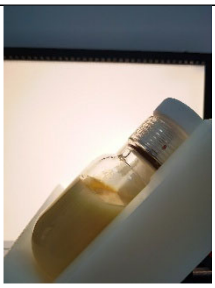
8h	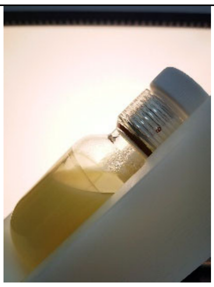	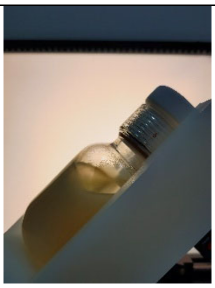	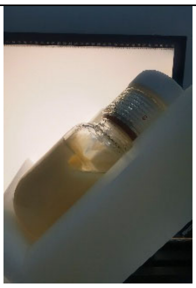	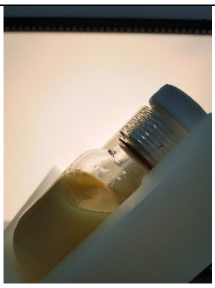	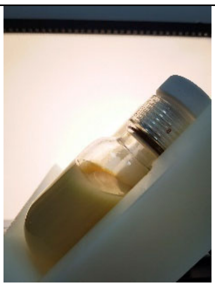

10h	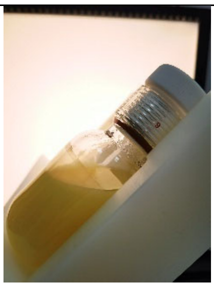	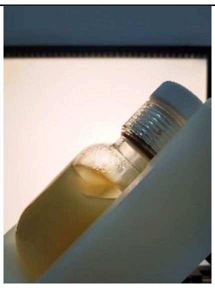	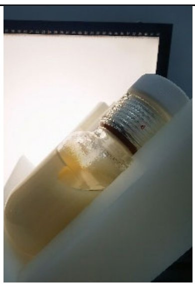	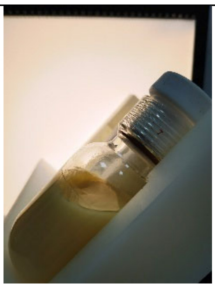	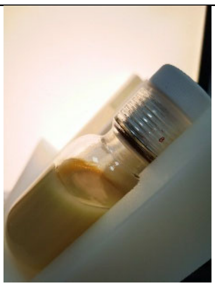
12h	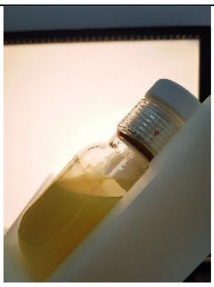	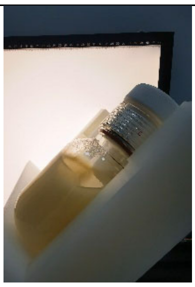	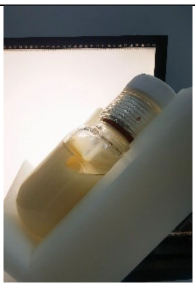	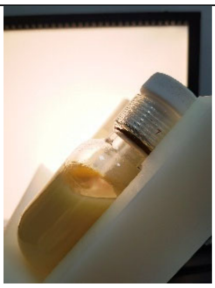	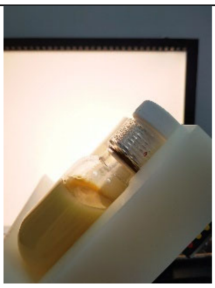
24h	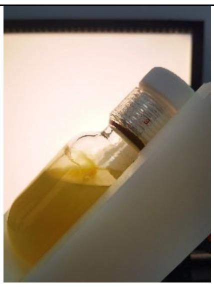	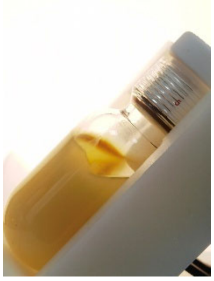	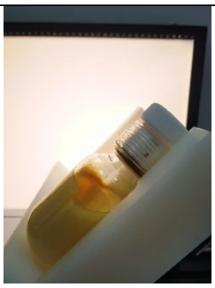	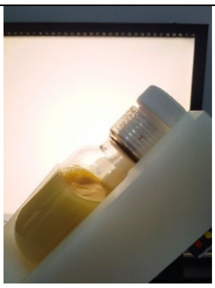	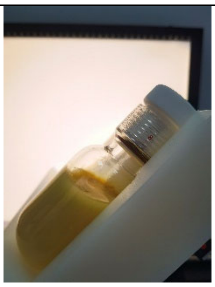
32h	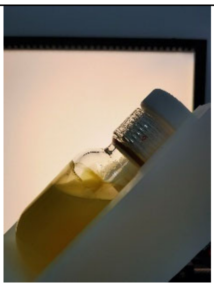	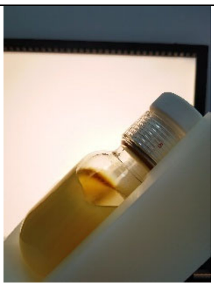	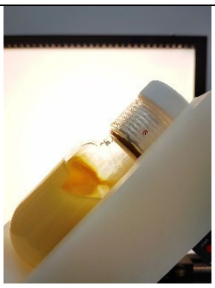	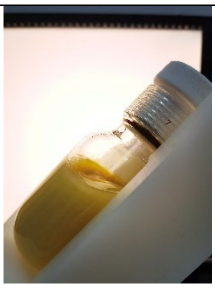	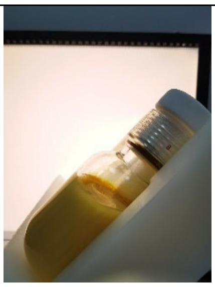
48h	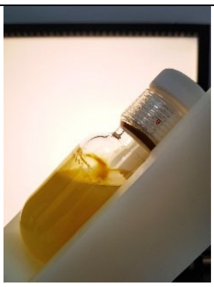	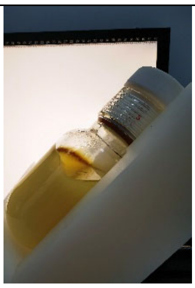	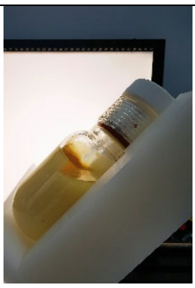	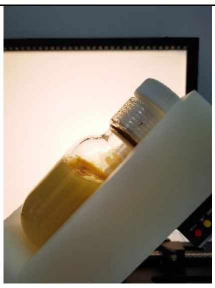	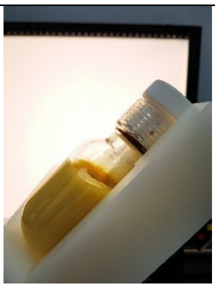

	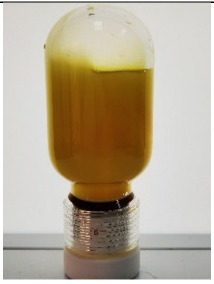	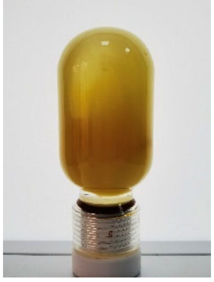	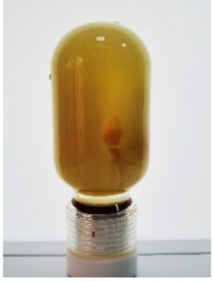	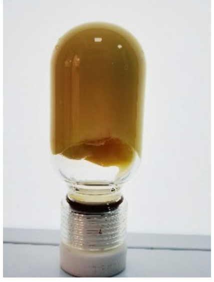	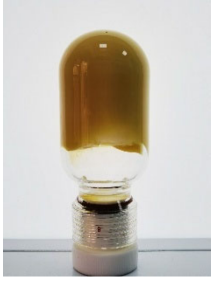
GS	C	D	E	F	G

Table S2. Optical images (at a fixed angle of 45° and upside down) during gelation process, at the SS content of 0.3% and at 130℃.

SS content / Time	0.3%	
0h	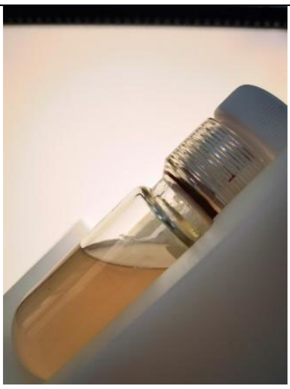	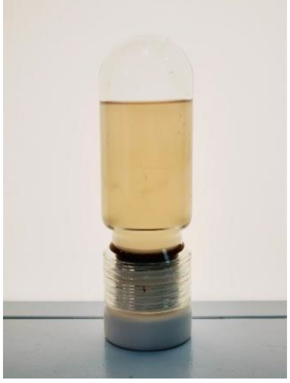
2h	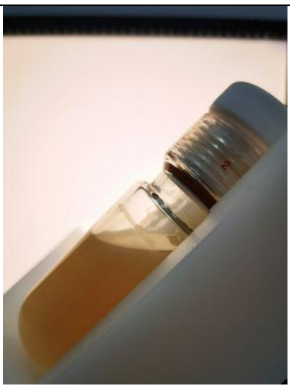	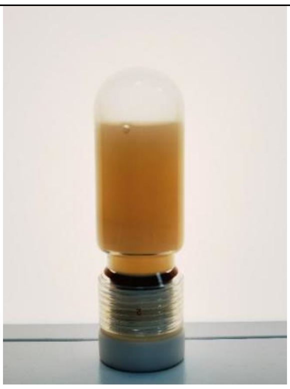
4h	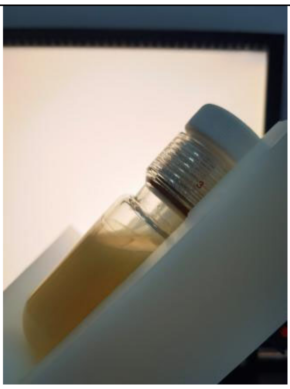	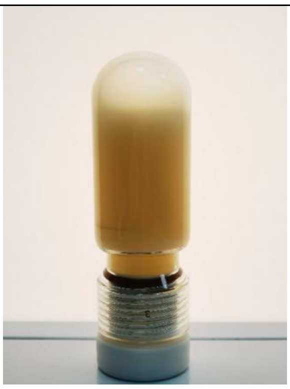

6h	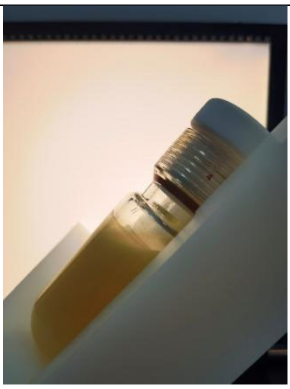	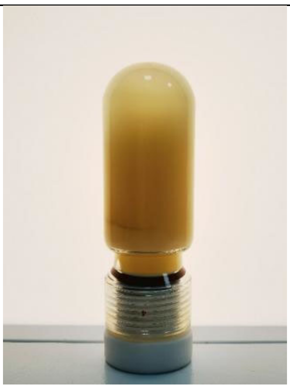
8h	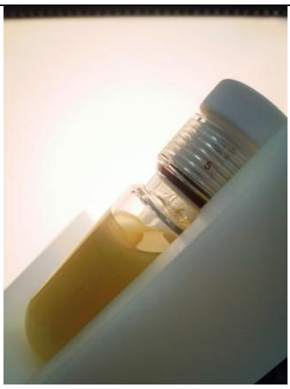	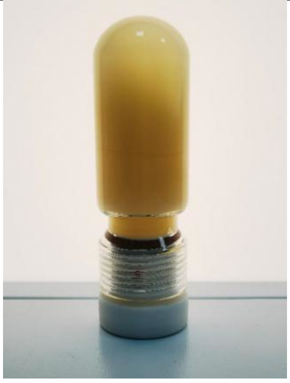
10h	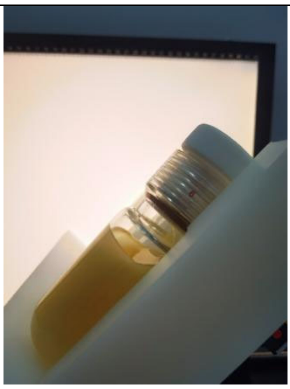	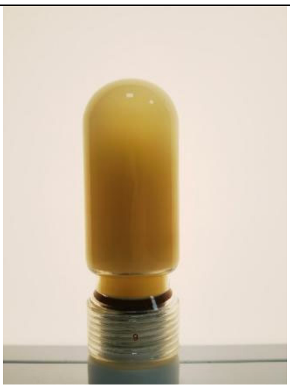
12h	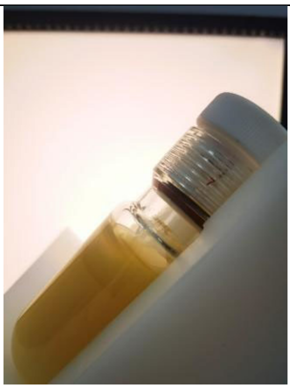	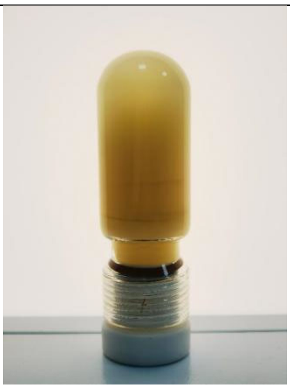

24h	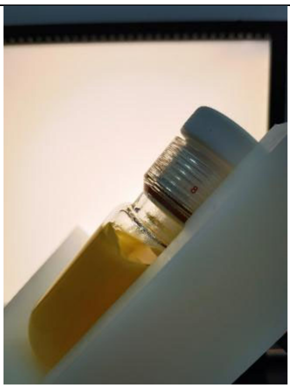	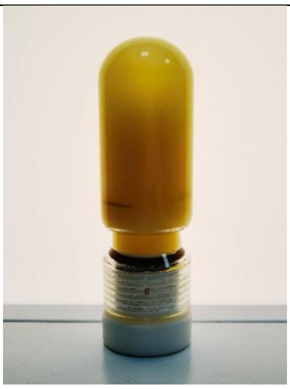
48h	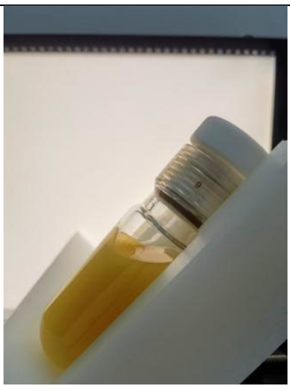	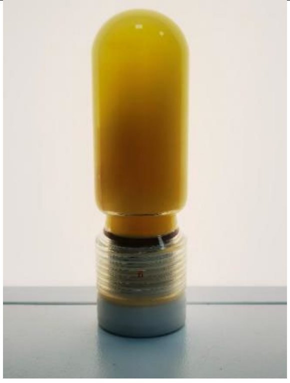
72h	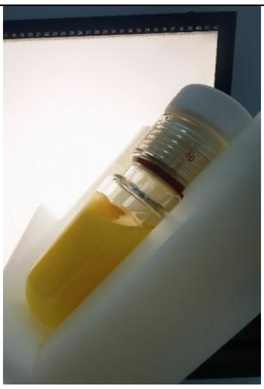	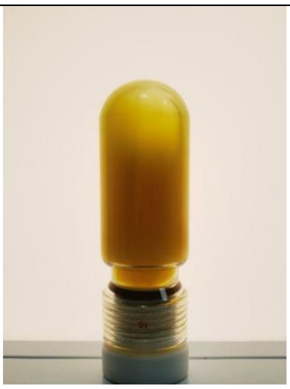
96h	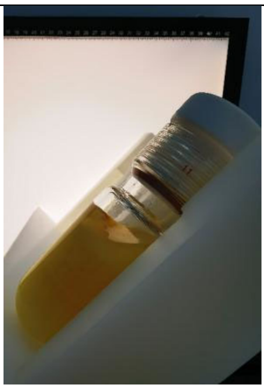	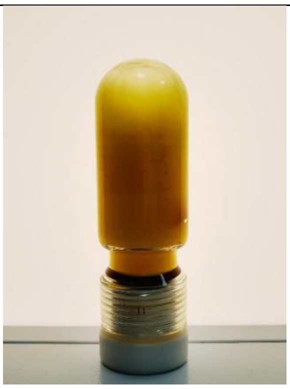

168h	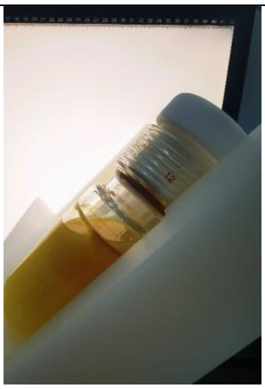	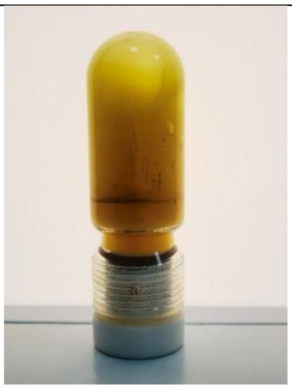
